# α-klotho reduces susceptibility to osteoarthritis: evidence from cross-sectional studies and Mendelian randomization

**DOI:** 10.3389/fendo.2024.1450472

**Published:** 2024-11-19

**Authors:** Zhao Li, Zhong Li, Qisheng Cheng, Xinlin Nie, Yu Cui, Bing Du, Taotao Ren, Yibo Xu, Teng Ma

**Affiliations:** ^1^ Honghui Hospital, Xi’an Jiaotong University, Xi’an, China; ^2^ Department of Orthopedic Center, The First Hospital of Jilin University, Changchun, China

**Keywords:** α-klotho, osteoarthritis, NHANES, Mendelian randomization, causal effect

## Abstract

**Background:**

Despite extensive research, the association between serum α-klotho levels and osteoarthritis (OA) remains unclear, predominantly relying on findings from OA mouse models. This study used data from the National Health and Nutrition Examination Survey (NHANES) to conduct a cross-sectional study examining the relationship between α-klotho and human OA. In addition, we used Mendelian randomization (MR) to genetically infer a causal relationship between serum α-klotho and the three OA subtypes.

**Method:**

A cohort of 12,037 subjects from NHANES (2007-2016) was analyzed. Multivariate logistic regression was utilized to examine the association between α-klotho concentration and OA, alongside subgroup analysis and interaction tests. Additionally, a two-sample bi-directional MR analysis was conducted to evaluate the relationship between serum α-klotho and three OA subtypes, including all OA, hip OA, and knee OA, employing the inverse variance weighting (IVW) method as the primary approach.

**Results:**

Following adjustment for covariates, a nonlinear negative correlation between serum α-klotho and OA was observed (OR=0.77; 95% CI, 0.68-0.88, p < 0.0001). The IVW method revealed that higher serum α-klotho levels were associated with decreased susceptibility to hip OA (OR = 0.92, 95% CI: 0.87–0.98, P = 9.64×10^-3^). However, MR analysis did not establish a causal relationship between serum α-klotho and OA or knee OA. Inverse MR also indicated that the three subtypes of OA do not causally affect serum α-klotho concentrations.

**Conclusions:**

In cross-sectional studies, α-klotho showed a nonlinear negative correlation with OA. MR analysis of outcomes was not identical to cross-sectional studies.

## Introduction

Osteoarthritis (OA) stands as a significant global contributor to pain and disability, affecting an estimated 10% of men and 18% of women over 60 years of age (1), imposing substantial societal and individual economic burdens (1, 2). There are various risk factors for OA such as age, female gender, genetic predisposition, comorbidities, obesity, and joint injuries (1, 3–8). OA was once thought to be a purely mechanical disease of cartilage degeneration, but it is now known to be a complex disease affecting the entire joint: cartilage, subchondral bone, synovium, and infrapatellar fat pad may all play a role in the pathogenesis of the disease, and it may be associated with systemic inflammation (1, 9–12). Biological pathways within joints are mechanosensitive (13). Prolonged mechanical stress and age-related wear and tear typically lead to cartilage degradation (14). But a breakthrough in non-surgical OA treatment efficacy remains elusive, underscoring the growing importance of investigating OA progression mechanisms (15).

The intricate molecular mechanisms underpinning OA development involve proteomics as a pivotal player (16, 17). Encoded by the Klotho gene, α-Klotho was initially characterized as an anti-aging protein (18). Its functions include inhibiting insulin and Wnt signaling pathways, mitigating oxidative stress, and regulating phosphatase activity and calcium uptake (18). Soluble α-klotho has paracrine and endocrine functions in different organs, and reduced levels of α-klotho in animal models of aging correlate with the initial manifestations of aging (18). Recent research has unveiled the role of α-Klotho’s epigenetic regulation in modulating the extracellular matrix associated with aging, thereby influencing chondrocyte physiology (19). Gu et al. noted a significant elevation of Klotho expression in normal mouse cartilage compared to OA models, alongside heightened activity in the Wnt/β-catenin pathway and its downstream targets in the latter (20). Similarly, Klotho gene transfer into mouse joints impeded cartilage degradation in OA models by suppressing the NOS2 and ZIP8-MMP13 catabolic axes, highlighting its crucial role in preserving cartilage tissue homeostasis (21). However, many existing mouse models of OA exhibit limitations; notably, they predominantly utilize post-traumatic young male mice, diverging from the predominantly age-related nature of human OA (19, 22). Furthermore, there remains uncertainty regarding the translatability of these preclinical models to human disease. Additionally, observational studies encounter inherent challenges in controlling for confounding factors and addressing bias from reverse causation, posing obstacles to elucidating the relationship between α-klotho and OA.

Mendelian randomization (MR) represents an innovative approach in genetic epidemiology, leveraging genome-wide association studies (GWAS) datasets and employing genetic variation as instrumental variables (IVs) to discern the causal effects of exposure on disease outcomes (23). By virtue of its random allocation and stability across germline genotypes, MR mitigates biases stemming from confounding and reverse causation (23, 24). In our current investigation, we initially utilized data sourced from the National Health and Nutrition Examination Survey (NHANES) to scrutinize the association between serum α-klotho concentrations and OA, adjusting for various potential confounders. Subsequently, we delved deeper into elucidating the causal link between serum α-klotho concentration and OA through MR analysis. From those, we wished to validate the effect of changes in human serum α-klotho concentration on OA susceptibility through a combination of observational and MR analyses.

## Method

All cross-sectional and GWAS data used in this study were appropriately consented by the participants and ethical approval was obtained in accordance with national legal and institutional requirements.

### Study design

In this study, we first examined the relationship between α-klotho and OA using data from the NHANES. We then analyzed summary statistics from GWAS using MR analysis and assessed the bidirectional causal associations between α-klotho and three osteoarthritis subtypes.

### Cross-sectional study

#### Description of data sources

**Figure 1:**
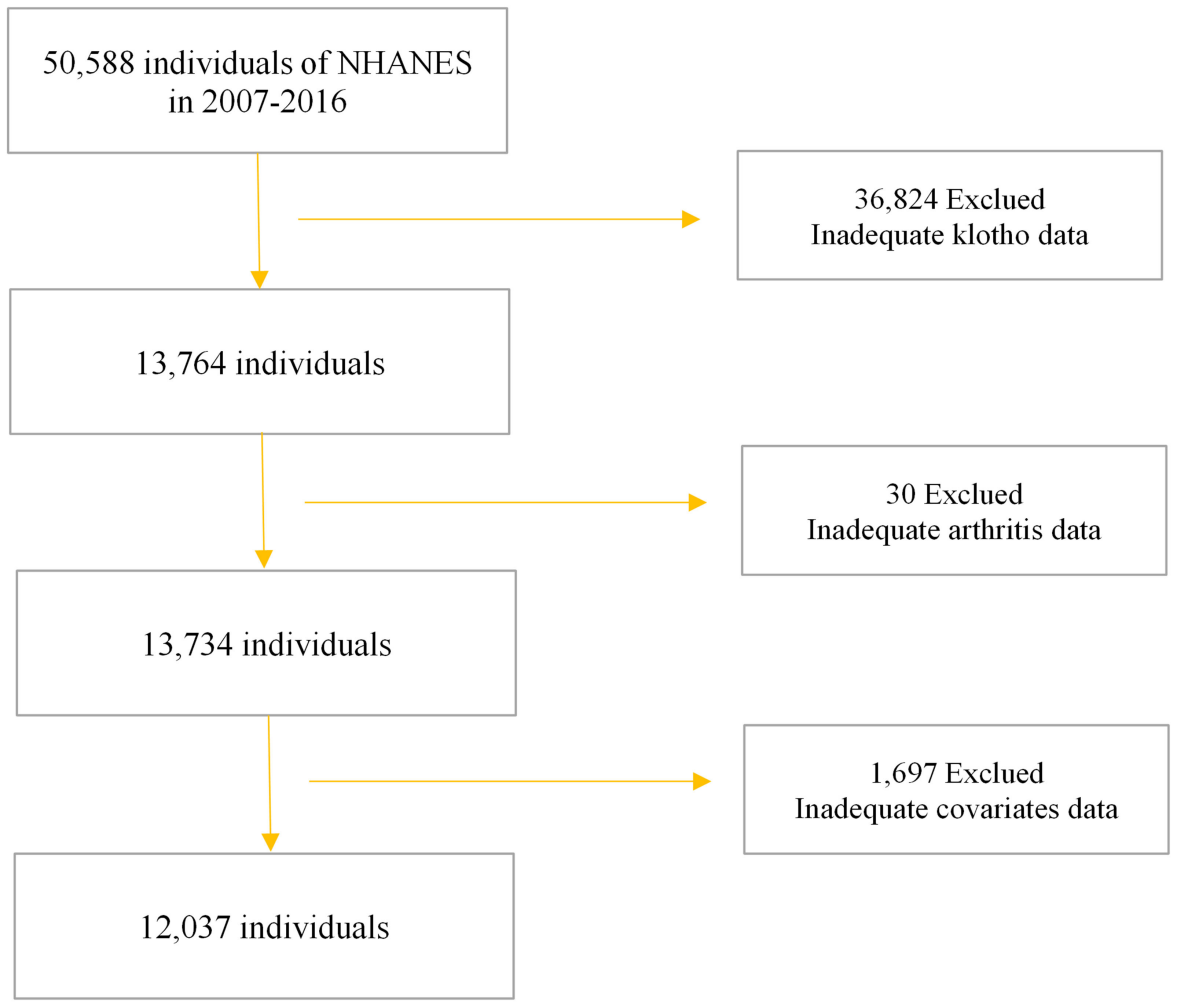
Flow chart of study participants.

The NHANES, conducted by the Centers for Disease Control and Prevention (CDC), serves as a comprehensive survey of the noninstitutionalized U.S. civilian population, ensuring national representation. The NHANES study protocols received approval from the National Center for Health Statistics Institutional Review Board (IRB), as documented at https://www.cdc.gov/nchs/nhanes/irba98.htm. Prior to participation, all individuals provided both verbal and written consent for their involvement in future research endeavors. Detailed information regarding interviews, medical examinations, and laboratory tests conducted during NHANES is accessible at www.cdc.gov/nchs/nhanes/. Our investigation utilized NHANES data spanning from 2007 to 2016, encompassing a cohort of 50,588 subjects. Following the exclusion of individuals with missing data on serum α-Klotho levels, OA, and covariates, our analysis involved a total of 12,037 participants (**Figure 1**).

#### OA defined

Participants with OA were identified based on a documented diagnosis by a healthcare professional. Individuals aged 20 years or older were queried with two specific questions: “Have you ever received a diagnosis of arthritis from a doctor or other healthcare professional?” and “If so, what type of arthritis were you diagnosed with?” Those respondents affirming both questions by indicating a diagnosis of osteoarthritis were classified into the OA group, while those providing different responses were categorized into the non-OA group(www.cdc.gov/nchs/nhanes/).

#### Serum α-klotho defined

Serum α-klotho served as the primary variable under observation in this study. Soluble α-klotho, a multifaceted protein encompassing paracrine and endocrine functions across various organs, was the focus of investigation. Diminished levels of α-klotho in animal aging models correlate with initial manifestations of aging. Klotho concentration was quantified in NHANES frozen samples using a commercially available enzyme-linked immunosorbent assay (ELISA) kit manufactured by IBL International, Japan (25). The samples were received on dry ice and were scanned in the laboratory receiving area where the data were compared to data received on an electronic manifest and then entered into the laboratory information system. Samples were analyzed in duplicate and averaged to calculate the final value. Results are automatically transferred from the instrument to the laboratory’s Oracle management system and are evaluated by the regional supervisor. Samples with more than 10% duplicate results are flagged for repeat analysis. If the QC sample value is not within 2SD of the specified value, the entire analytical run is rejected and the sample is repeated.

### Covariates

Covariates encompassed demographic data such as age, gender, and race. Body mass index (BMI) was assessed by healthcare professionals at the Mobile Examination Center (MEC). Diabetes and hypertension were delineated based on documented physician diagnoses or prescribed medications. Cotinine concentrations served as indicators of both active smoking and exposure to secondhand smoke. Individuals classified as alcohol drinkers were those who reported consuming more than 12 alcoholic beverages within the past year.

#### Statistical

The statistical analyses were carried out employing Empower Stats (version 2.0). To compare the demographic characteristics of subjects in the OA and non-OA groups, t-tests and chi-square tests were utilized. Beta values and their corresponding 95% confidence intervals were computed using multivariate logistic regression analysis to assess the relationship between α-klotho concentration and OA.

Model 1 was unadjusted, Model 2 incorporated adjustments for age, race, and gender, while Model 3 included additional adjustments for age, gender, race, education level, Diabetes, Hypertension, Alcohol drinking, Cotinine, and BMI. Simultaneous smoothed curve fits were performed after adjusting these variables. Furthermore, subgroup analyses and interaction tests were also undertaken to investigate the relationship between the α-klotho concentration and OA. We analyzed subgroups separately for BMI, gender, and age. Subjects were divided into three groups according to BMI <25, 25-30, and >30; two groups according to gender; and two groups according to age 40-59 and 60-79 for subgroup analyses, respectively, in order to further exclude confounding factors. The threshold for statistical significance was defined as p < 0.05.

### Mendelian randomization

**Figure 2:**
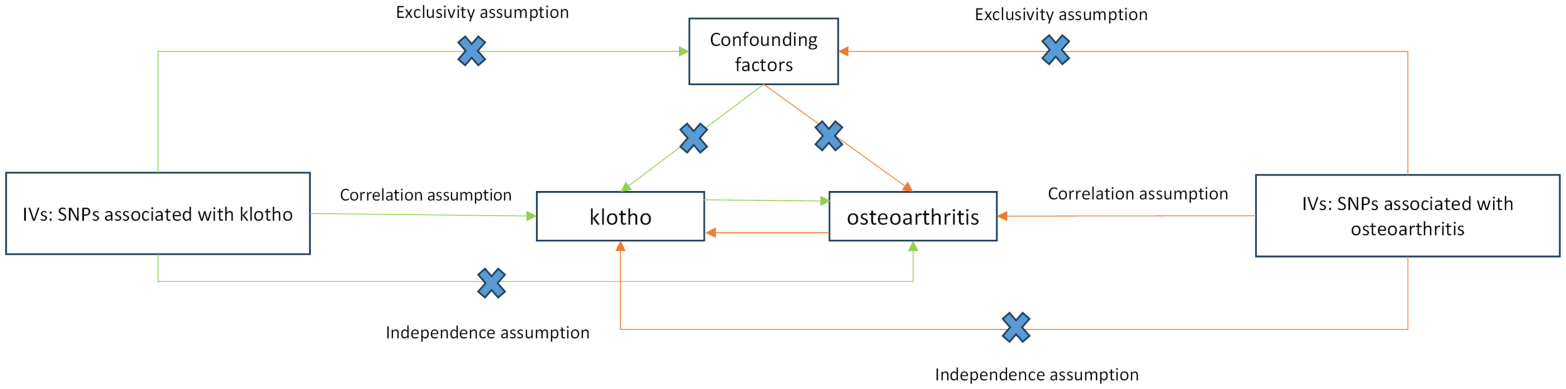
Schematic representation of Mendelian randomization analysis. α-klotho and osteoarthritis as exposure and outcome, respectively, and the instrumental variables must meet three major assumptions. IVs, instrument variables; SNPs, single-nucleotide polymorphisms.

**Figure 2** presents a flowchart elucidating the bidirectional causal relationship between α-klotho and OA. For an MR study to yield valid results, three key assumptions must be met (1): genetic variants must exhibit correlation with the exposure under investigation (2); these variants should not be linked with confounding factors; and (3) they should solely influence the outcome through the exposure. To conduct bidirectional two-sample MR analyses, we constructed distinct IVs comprising independent single nucleotide polymorphisms (SNPs) associated with α-klotho and the three subtypes of OA, including all OA, knee OA and hip OA.

### Data source

A summary-level GWAS dataset including data for α-klotho was extracted from a comprehensive meta-analysis of circulating α-klotho (26), comprising 4,675 European-ancestry participants. The GWAS data for OA consisted of 39,515 cases and 445,083 controls, and all subjects were from UK ancestry (27). OA is defined as a non-inflammatory degenerative joint disease occurring primarily in older adults and characterized by degeneration of articular cartilage, marginal osteophytes and synovial changes. It is associated with pain and stiffness, especially after prolonged activity (27). To increase the generalizability of the study, we additionally added two major OA subtypes, knee OA and hip OA. Pooled GWAS data for these two phenotypes were obtained from UK biobank, containing 403,124 and 393,873 UK ancestry individual data, respectively (28).

### Instrumental variables extracting

We conducted SNP screening based on genome-wide significance (p < 5 × 10^−8^) regarding their correlation with the exposure, followed by further refinement through the elimination of linkage disequilibrium (LD) (clumping r2 = 0.001 and kb = 10,000) to identify instrumental variables (IVs) (29). Subsequently, prior to each MR analysis, IVs associated with the outcome were excluded. Additionally, we utilized PhenoScanner (www.phenoscanner.medschl.cam.ac.uk), an extensive platform providing detailed information on genotype-phenotype relationships, to identify and eliminate potential confounders by screening out secondary phenotypes of SNPs.

#### Statistical

The inverse-variance weighted (IVW) method (30) served as our primary MR analytical approach. To determine the causal association between OA and α-klotho, we conducted random-effects meta-analysis, combining the ratios of SNP-exposure to SNP-outcome. Additionally, to enhance the precision of IVW estimates, we employed MR-Egger and weighted median methods. For sensitivity analysis, we utilized Cochran’s Q test to assess heterogeneity. Furthermore, we employed the MR-Egger intercept test, “leave-one-out analyses,” funnel plot, and MR-PRESSO method to evaluate horizontal and directional pleiotropy.

All statistical analyses were performed using the TwoSampleMR packages in R software (Version 4.3.0), and p-value < 0.05 was seen as significantly association.

## Results

### Cross-sectional study

In the cross-sectional study, a total of 12,036 participants were included, of which 48.85% were males and 51.15% were females, with a mean age of 57.86 ± 10.81 years. Among all the participants, 1,686 (14%) had OA. Age, gender, race, education, cotinine, BMI, diabetes, hypertension, and serum α-klotho concentrations were statistically significant between OA and non-OA individuals (p < 0.05). OA patients, compared to those without, were more likely to be female, Non-Hispanic White, be nonsmoker, exhibit advanced age, higher BMI, in our study (p < 0.05). **Table 1** presents the clinical and biochemical characteristics of the subjects based on whether they were OA patients.

**Table 1:** Clinical characteristics of all 12,036 subjects among subjects with OA and without OA.

Characteristics	Control	OA	*P*-value
	n=10,350	n=1,686	
Age, years	57.07 ± 10.76	62.75 ± 9.79	<0.001
Gender (%)			<0.001
Male	50.79	36.95	
Female	49.21	63.05	
Race (%)			<0.001
Mexican American	16.38	9.49	
Other Hispanic	11.50	7.59	
Non-Hispanic White	42.42	61.21	
Non-Hispanic Black	20.46	15.95	
Other Race	9.25	5.75	
Education level (%)			<0.001
<9th grade	13.19	8.13	
9–11th grade	14.91	11.27	
High school grade/GED or equivalent	22.49	21.17	
Some college or AA degree	27.02	31.49	
College graduate or above	22.39	27.94	
Diabetes (%)			<0.001
Yes	17.55	20.76	
No	79.68	75.09	
Borderline	2.77	4.15	
Hypertension (%)			<0.001
Yes	45.05	59.85	
No	54.95	40.15	
Alcohol drinking (%)			0.809
Yes	71.00	71.29	
No	29.00	28.71	
Cotinine, (ng/mL)	61.27 ± 136.18	52.37 ± 124.88	<0.012
BMI, kg/m2	29.57 ± 6.51	31.63 ± 7.88	<0.001
α-klotho, (pg/ml)	857.43± 312.98	821.54± 284.50	<0.001

Mean ± SD for continuous variables: the P value was calculated by weighted linear regression model.

OA, osteoarthritis; BMI, body mass index.

**Figure 3:**
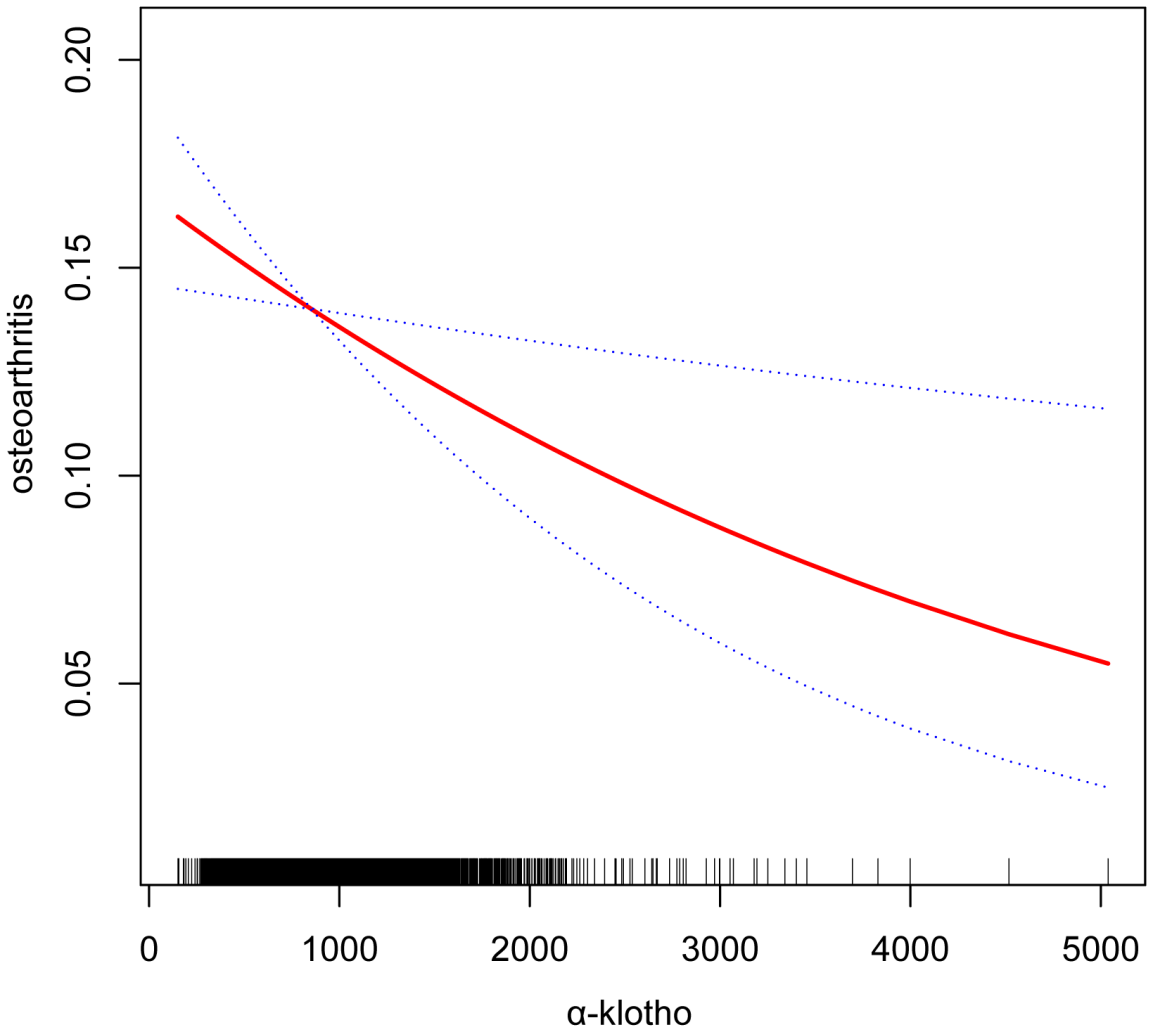
Smooth curve fittings. Association between α-klotho and osteoarthritis, Gender, Age, Race, Education level, Diabetes, Hypertension, Alcohol drinking, Cotinine, and BMI were adjusted; The solid and dotted lines represent the estimated values and their corresponding 95% CIs, respectively.

Our study reveals an association between elevated serum α-klotho concentrations and susceptibility to OA. In the unadjusted model, a 1ug/ml increase in α-klotho concentration was associated with a 34% reduction in the risk of OA development (OR=0.66; 95% CI, 0.55-0.80, p < 0.0001). Similarly, in the minimally adjusted model, each 1ug/ml increase in α-klotho concentration was associated with a 24% reduction in susceptibility to OA (OR=0.76; 95% CI, 0.64-0.92, p = 0.0047). To further verify the robustness of the conclusions, we triple α-klotho and perform a sensitivity analysis. Individuals in the highest tertile of α-klotho concentration had a statistically significant 23% lower risk of developing OA compared to individuals in the lowest tertile of α-klotho concentration (OR=0.77; 95% CI, 0.68-0.88, p < 0.0001). In addition, participants in the middle third of the α-klotho concentration group also exhibited a lower risk of OA compared to the lowest third (OR=0.83; 95% CI, 0.73-0.94, p=0.0034) (**Table 2**). In addition, an attempt was made to fit the nonlinear relationship between α-klotho concentration and OA into a smooth curve, which showed a nonlinear negative correlation between the two variables (**Figure 3**).

**Table 2:** The associations between α-klotho and OA.

Exposure	Model I OR (95% CI) *P*	Model II OR (95% CI) *P*	Model III OR (95% CI) *P*
α-klotho	0.66 (0.55, 0.80) <0.0001	0.76(0.64, 0.92) 0.0047	0.77 (0.63, 0.93) 0.0061
Klotho classification			
Lowα-klotho (<0.7014)	Reference	Reference	Reference
Middleα-klotho (0.7014-0.9152)	0.83 (0.73, 0.94) 0.0034	0.88 (0.78, 1.00) 0.0541	0.87 (0.77, 0.99) 0.0392
Highα-klotho (>0.9152)	0.77 (0.68, 0.88) <0.0001	0.85 (0.75, 0.97) 0.0166	0.85 (0.74, 0.97) 0.0154
*P* for trend	0.60 (0.47, 0.78) <0.0001	0.73 (0.56, 0.95) 0.0194	0.73 (0.56, 0.95) 0.0185

Model I: None covariates were adjusted;

Model II: Gender, Age, and Race were adjusted;

Model III: Gender, Age, Race, Education level, Diabetes, Hypertension, Alcohol drinking, Cotinine, and BMI were adjusted.

OA, osteoarthritis; BMI, body mass index.

We analyzed subgroups separately for BMI, gender, and age. As shown in **Table 3**, there was no significant correlation between the three subgroups of BMI and the interaction test between the two gender subgroups (P for interaction>0.05). This means that there is no evidence that the association between α-klotho and OA is related to BMI and gender. Notably, when we divided the age into two subgroups, 40-59 and 60-79, for the interaction test, the results suggested that the association between α-klotho and OA might have different effects in different age groups (p=0.031).

**Table 3:** Subgroup analysis for the association between α-klotho and OA.

Subgroup	OR (95%CI)	*P* for interaction
BMI		0.870
<25	0.68 (0.46, 1.02) 0.0592	
25-30	0.62 (0.44, 0.88) 0.0067	
≥30	0.70 (0.54, 0.90) 0.0056	
Gender		0.964
Male	0.61 (0.45,0.84) 0.0025	
Female	0.62 (0.50,0.78) <0.0001	
Age		0.031
40-59	0.56 (0.41,0.77) 0.0003	
60-79	0.85 (0.68,1.07) 0.1635	

In the subgroup analyses, the model is not adjusted for the stratification variable itself.

OA, osteoarthritis; BMI, body mass index.

### Mendelian randomization

Ultimately, we identified 5 IVs for genetic prediction of circulating α-klotho. Additionally, 7 index SNPs were selected for genetic prediction of overall OA, with 10 index SNPs designated for knee OA and 27 index SNPs for hip OA, representing the two OA subtypes. Detailed IVs information is provided in the **Supplementary Table S1–S4**. Subsequently, we conducted a bidirectional two-sample MR study to elucidate the causal relationship between circulating α-klotho and OA.

**Figure 4:**
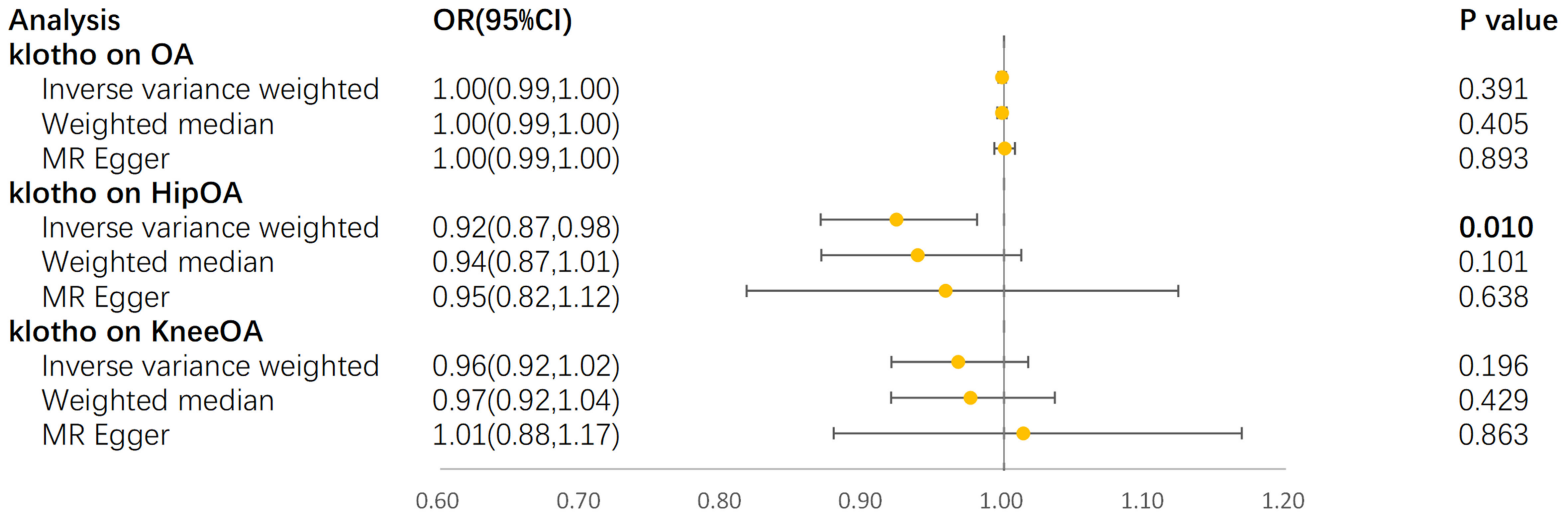
Forest plot for MR analyses. OR, odds ratio; CI, confidence interval; IVW, Inverse-variance weighted; OA, osteoarthritis.

In the IVW model, α-klotho demonstrated a significant reduction in the risk of hip OA (OR = 0.92, 95% CI: 0.87–0.98, P = 9.64×10^-3^) (**Figure 4**). The Cochran’s Q-derived P-value of 0.56 suggested no evidence of heterogeneity, while the MR-Egger intercept-derived P-value of 0.66 indicated no discernible pleiotropy. Conversely, no causal association between α-klotho and overall OA or knee OA was observed. Furthermore, in the reverse MR analysis, no significant causal effect of the three OA subtypes on circulating α-klotho was evident. The results of all sensitivity analyses are presented in the **Supplementary Figure S1**.

## Discussion

This study provides a comprehensive analysis of the relationship between human serum α-klotho protein and OA using data from a large observational study and an extensive genetic dataset. Investigations across diverse clinical populations have indicated a nonlinear negative correlation between serum α-klotho levels and OA, suggesting a potential protective role for α-klotho in cartilage health. Notably, subgroup analyses conducted across different age groups revealed varying associations between α-klotho concentration and OA, may indicating age-dependent effects. Xu et al. performed similar work and came to conclusions similar to ours (31). In our study, we further utilized MR analysis to stratify subgroups of OA on top of conducting observational studies, and investigated the relationship between klotho and susceptibility to the three OA subtypes at the genetic level. MR studies have further highlighted a significant protective effect of increased serum α-klotho concentration specifically in individuals with the hip OA subtype. However, no significant associations were observed between serum α-klotho levels and overall OA or knee OA. Additionally, reverse MR analysis did not suggest a causal effect of the three OA subtypes on serum α-klotho levels. These findings provide valuable insights into the mechanisms underlying OA and aging processes, shedding light on the potential role of serum α-klotho in these contexts.

The *Klotho* (KL) gene, initially identified as a potential senescence suppressor gene, has sparked significant interest due to its impact on lifespan regulation (32–34). Mouse studies have shown that overexpression of the KL gene extends lifespan, while mutations in this gene shorten it, thereby deepening our understanding of the aging process (35, 36). The human KL gene encodes the multifunctional α-Klotho protein, which plays a crucial role in regulating phosphate, calcium, and vitamin D metabolism (18). Although research on α-klotho and OA is relatively limited, existing findings consistently suggest its relevance. Notably, dating back to 2007, Zhang et al. examined the association between α-klotho gene expression and OA susceptibility in a female Caucasian population, observing an association between a *klotho* gene variant and hand OA susceptibility (37). In recent years, numerous mouse models of OA have reinforced the notion that α-klotho serves as a protective factor against OA progression (20, 21). For instance, Gu et al. demonstrated that Klotho acts as an antagonist to endogenous Wnt/β-catenin activity, thereby mitigating cartilage damage in OA (20). Furthermore, studies in rat models have shown that treatment with α-klotho and sTGFβR2 counteracts the development of OA phenotype, offering potential avenues for future cartilage repair drug development (38). Additionally, investigations by Iijima examining OA-related changes in mice across lifespan and gender found that age-related alterations in extracellular matrix properties initiate pathogenic mechanotransduction signals, leading to α-klotho promoter methylation and compromised cellular health, with broader implications for aging research (19). Despite the high degree of sequence similarity (98%) between human and mouse α-Klotho proteins (18), caution must be exercised in directly applying conclusions from animal models to humans. However, our study, based on data from a large-scale human observational study, reaffirms previous findings suggesting that higher serum α-klotho concentrations may reduce susceptibility to OA. This underscores a potential avenue for future OA prevention and non-surgical cartilage treatment strategies.

In contrast to the cross-sectional findings, MR analysis only indicated a protective effect of serum α-klotho causality on the hip OA subtype. The absence of heterogeneity and pleiotropy in the sensitivity analyses, including MR-Egger, Cochran’s Q test, and leave-one-out sensitivity, underscores the reliability of these positive findings. Furthermore, to mitigate the possibility of reverse causation, we conducted bidirectional MR analysis (39). However, MR results did not suggest a causal association between serum α-klotho concentrations and the other two OA subtypes. This disparity from observational studies may arise from the fact that participants in this cross-sectional study were exclusively middle-aged and older adults aged over 50 years, whereas MR analyses estimated exposure effects over a lifetime. It remains unclear whether an increase in serum α-klotho can exclusively reduce susceptibility to OA during specific periods of time, a bias observed in previous klotho studies (40). However, it is imperative to note that MR analysis provides a higher level of evidence. Consequently, we cannot infer that elevated serum α-klotho concentrations would significantly protect against all OA subtypes.

Our study boasts several strengths. Firstly, our cross-sectional investigation benefits from a sizable sample size and employs a diverse array of statistically robust methodologies. Secondly, we conducted a comprehensive two-sample MR analysis to assess the causal relationship between serum α-klotho and various subtypes of OA phenotypes. In contrast to observational studies, MR analysis offers the advantage of mitigating potential confounders and reverse causality. Thirdly, while prior research predominantly relied on mouse OA models, our study utilized human data, thus enhancing the reliability of our conclusions. However, our study also exhibits several limitations. Firstly, our MR analysis only considered a linear relationship between exposure and outcome, thereby failing to account for potential nonlinear relationships. Secondly, due to limitations in the available GWAS data, we were unable to conduct stratified MR analysis by sex, age, OA grade, classification and other factors, complicating the reconciliation of discrepancies with cross-sectional study findings. Third, whether subjects had OA in our observational study came from self-report rather than clinical diagnosis, and thus may have been partially biased. Fourth, our study analyses used only cross-sectional data from the U.S. population and GWAS data from individuals of European ancestry, which limits the generalizability of our findings to all population groups. Future analyses should incorporate data from different ethnic backgrounds to improve the applicability of our findings to different populations. Finally, our study focused solely on investigating the association between serum α-klotho and OA susceptibility, without delving into the specific underlying mechanisms. Further basic research and animal experiments are warranted to elucidate these mechanisms in greater detail.

## Conclusion

Our cross-sectional study found an inverse nonlinear relationship between serum α-klotho and OA. MR analyses revealed that the conclusions of the cross-sectional study also applied to serum α-klotho and hip OA subtypes in European pedigrees. However, OA and knee OA subtypes were not found to be causally related to klotho. It is possible that klotho may not have the same effect on OA in different age strata, which may also account for the discrepancy between the findings of MR analyses and cross-sectional studies.

## Data Availability

The original contributions presented in the study are included in the article/**Supplementary Material**. Further inquiries can be directed to the corresponding authors.
