# Supplementary material for: α-klotho reduces susceptibility to osteoarthritis: evidence from cross-sectional studies and Mendelian randomization
Source: Front Endocrinol (Lausanne). 2024 Nov 19;15:1450472. doi: 10.3389/fendo.2024.1450472 (PMC11611571; doi:10.3389/fendo.2024.1450472)
Supplement: Supplementary file 1 [file DataSheet1.docx]

**STROBE-MR checklist of recommended items to address in reports of Mendelian randomization studies**^1^ ^2^

| **Item No.** | **Section** | **Checklist item** | **Relevant text from manuscript** |
| --- | --- | --- | --- |
| 1 | **TITLE and ABSTRACT** | Indicate Mendelian randomization (MR) as the study’s design in the title and/or the abstract if that is a main purpose of the study | Association of α-klotho and osteoarthritis: A cross-sectional study and Mendelian randomization study |
|  | **INTRODUCTION** |  |  |
| 2 | **Background** | Explain the scientific background and rationale for the reported study. What is the exposure? Is a potential causal relationship between exposure and outcome plausible? Justify why MR is a helpful method to address the study question | Observational studies encounter inherent challenges in controlling for confounding factors and addressing bias from reverse causation, posing obstacles to elucidating the relationship between α-klotho and OA. |
| 3 | **Objectives** | State specific objectives clearly, including pre-specified causal hypotheses (if any). State that MR is a method that, under specific assumptions, intends to estimate causal effects | Mendelian randomization (MR) represents an innovative approach in genetic epidemiology, leveraging genome-wide association studies (GWAS) datasets and employing genetic variation as instrumental variables (IVs) to discern the causal effects of exposure on disease outcomes. Subsequently, we delved deeper into elucidating the causal link between serum α-klotho concentration and OA through MR analysis. |
|  | **METHODS** |  |  |
| 4 | **Study design and data sources** | Present key elements of the study design early in the article. Consider including a table listing sources of data for all phases of the study. For each data source contributing to the analysis, describe the following: |  |
|  | a) | Setting: Describe the study design and the underlying population, if possible. Describe the setting, locations, and relevant dates, including periods of recruitment, exposure, follow-up, and data collection, when available. | A summary-level GWAS dataset including data for α-klotho was extracted from a comprehensive meta-analysis of circulating α-klotho, comprising 4,675 European-ancestry participants. The GWAS data for OA consisted of 39,515 cases and 445,083 controls, and all subjects were from the UK ancestry |
|  | b) | Participants: Give the eligibility criteria, and the sources and methods of selection of participants. Report the sample size, and whether any power or sample size calculations were carried out prior to the main analysis | A summary-level GWAS dataset including data for α-klotho was extracted from a comprehensive meta-analysis of circulating α-klotho, comprising 4,675 European-ancestry participants. The GWAS data for OA consisted of 39,515 cases and 445,083 controls, and all subjects were from the UK ancestry |
|  | c) | Describe measurement, quality control and selection of genetic variants | We conducted SNP screening based on genome-wide significance (p < 5 × 10−8) regarding their correlation with the exposure, followed by further refinement through the elimination of linkage disequilibrium (LD) (clumping r2 = 0.001 and kb = 10,000) to identify instrumental variables (IVs) |
|  | d) | For each exposure, outcome, and other relevant variables, describe methods of assessment and diagnostic criteria for diseases | The inverse-variance weighted (IVW) method served as our primary MR analytical approach. To determine the causal association between OA and α-klotho, we conducted random-effects meta-analysis, combining the ratios of SNP-exposure to SNP-outcome. Additionally, to enhance the precision of IVW estimates, we employed MR-Egger and weighted median methods. For sensitivity analysis, we utilized Cochran's Q test to assess heterogeneity. Furthermore, we employed the MR-Egger intercept test, "leave-one-out analyses," funnel plot, and MR-PRESSO method to evaluate horizontal and directional pleiotropy. |
|  | e) | Provide details of ethics committee approval and participant informed consent, if relevant | Ethics approval and consent to participate  The data used in this paper are publicly available, ethically approved, and the subjects have given their informed consent. |
| 5 | **Assumptions** | Explicitly state the three core IV assumptions for the main analysis (relevance, independence and exclusion restriction) as well assumptions for any additional or sensitivity analysis | Figure 2 presents a flowchart elucidating the bidirectional causal relationship between α-klotho and OA. For a MR study to yield valid results, three key assumptions must be met: (1) genetic variants must exhibit correlation with the exposure under investigation; (2) these variants should not be linked with confounding factors; and (3) they should solely influence the outcome through the exposure. |
| 6 | **Statistical methods: main analysis** | Describe statistical methods and statistics used |  |
|  | a) | Describe how quantitative variables were handled in the analyses (i.e., scale, units, model) | We conducted SNP screening based on genome-wide significance (p < 5 × 10−8) regarding their correlation with the exposure, followed by further refinement through the elimination of linkage disequilibrium (LD) (clumping r2 = 0.001 and kb = 10,000) to identify instrumental variables (IVs) |
|  | b) | Describe how genetic variants were handled in the analyses and, if applicable, how their weights were selected | We conducted SNP screening based on genome-wide significance (p < 5 × 10−8) regarding their correlation with the exposure, followed by further refinement through the elimination of linkage disequilibrium (LD) (clumping r2 = 0.001 and kb = 10,000) to identify instrumental variables (IVs) |
|  | c) | Describe the MR estimator (e.g. two-stage least squares, Wald ratio) and related statistics. Detail the included covariates and, in case of two-sample MR, whether the same covariate set was used for adjustment in the two samples | The inverse-variance weighted (IVW) method served as our primary MR analytical approach. To determine the causal association between OA and α-klotho, we conducted random-effects meta-analysis, combining the ratios of SNP-exposure to SNP-outcome. Additionally, to enhance the precision of IVW estimates, we employed MR-Egger and weighted median methods. For sensitivity analysis, we utilized Cochran's Q test to assess heterogeneity. Furthermore, we employed the MR-Egger intercept test, "leave-one-out analyses," funnel plot, and MR-PRESSO method to evaluate horizontal and directional pleiotropy. |
|  | d) | Explain how missing data were addressed |  |
|  | e) | If applicable, indicate how multiple testing was addressed |  |
| 7 | **Assessment of assumptions** | Describe any methods or prior knowledge used to assess the assumptions or justify their validity | The inverse-variance weighted (IVW) method served as our primary MR analytical approach. To determine the causal association between OA and α-klotho, we conducted random-effects meta-analysis, combining the ratios of SNP-exposure to SNP-outcome. |
| 8 | **Sensitivity analyses and additional analyses** | Describe any sensitivity analyses or additional analyses performed (e.g. comparison of effect estimates from different approaches, independent replication, bias analytic techniques, validation of instruments, simulations) | Additionally, to enhance the precision of IVW estimates, we employed MR-Egger and weighted median methods. For sensitivity analysis, we utilized Cochran's Q test to assess heterogeneity. Furthermore, we employed the MR-Egger intercept test, "leave-one-out analyses," funnel plot, and MR-PRESSO method to evaluate horizontal and directional pleiotropy. |
| 9 | **Software and pre-registration** |  |  |
|  | a) | Name statistical software and package(s), including version and settings used | All statistical analyses were performed using the TwoSampleMR packages in R software (Version 4.3.0), and p-value < 0.05 was seen as significantly association. |
|  | b) | State whether the study protocol and details were pre-registered (as well as when and where) |  |
|  | **RESULTS** |  |  |
| 10 | **Descriptive data** |  |  |
|  | a) | Report the numbers of individuals at each stage of included studies and reasons for exclusion. Consider use of a flow diagram | Ultimately, we identified 5 IVs for genetic prediction of circulating α-klotho. Additionally, 7 index SNPs were selected for genetic prediction of overall OA, with 10 index SNPs designated for knee OA and 27 index SNPs for hip OA, representing the two OA subtypes |
|  | b) | Report summary statistics for phenotypic exposure(s), outcome(s), and other relevant variables (e.g. means, SDs, proportions) | Ultimately, we identified 5 IVs for genetic prediction of circulating α-klotho. Additionally, 7 index SNPs were selected for genetic prediction of overall OA, with 10 index SNPs designated for knee OA and 27 index SNPs for hip OA, representing the two OA subtypes |
|  | c) | If the data sources include meta-analyses of previous studies, provide the assessments of heterogeneity across these studies |  |
|  | d) | For two-sample MR:  i.  Provide justification of the similarity of the genetic variant-exposure associations between the exposure and outcome samples  ii.  Provide information on the number of individuals who overlap between the exposure and outcome studies | Exposure outcomes have been mentioned in the methodology as coming from different populations with no overlap |
| 11 | **Main results** |  |  |
|  | a) | Report the associations between genetic variant and exposure, and between genetic variant and outcome, preferably on an interpretable scale | In the IVW model, α-klotho demonstrated a significant reduction in the risk of hip OA (OR = 0.92, 95% CI: 0.87–0.98, P = 9.64×10^-3^) |
|  | b) | Report MR estimates of the relationship between exposure and outcome, and the measures of uncertainty from the MR analysis, on an interpretable scale, such as odds ratio or relative risk per SD difference | In the IVW model, α-klotho demonstrated a significant reduction in the risk of hip OA (OR = 0.92, 95% CI: 0.87–0.98, P = 9.64×10^-3^) |
|  | c) | If relevant, consider translating estimates of relative risk into absolute risk for a meaningful time period | If relevant, consider translating estimates of relative risk into absolute risk for a meaningful time period |
|  | d) | Consider plots to visualize results (e.g. forest plot, scatterplot of associations between genetic variants and outcome versus between genetic variants and exposure) | The results of all sensitivity analyses are presented in the Supplementary Figure S1. |
| 12 | **Assessment of assumptions** |  |  |
|  | a) | Report the assessment of the validity of the assumptions | Detailed IVs information is provided in the Supplementary Table S1-4. |
|  | b) | Report any additional statistics (e.g., assessments of heterogeneity across genetic variants, such as *I^2^*, Q statistic or E-value) |  |
| 13 | **Sensitivity analyses and additional analyses** |  |  |
|  | a) | Report any sensitivity analyses to assess the robustness of the main results to violations of the assumptions | The Cochran's Q-derived P-value of 0.56 suggested no evidence of heterogeneity, while the MR-Egger intercept-derived P-value of 0.66 indicated no discernible pleiotropy. |
|  | b) | Report results from other sensitivity analyses or additional analyses | The results of all sensitivity analyses are presented in the Supplementary Figure S1. |
|  | c) | Report any assessment of direction of causal relationship (e.g., bidirectional MR) | Furthermore, in the reverse MR analysis, no significant causal effect of the three OA subtypes on circulating α-klotho was evident. |
|  | d) | When relevant, report and compare with estimates from non-MR analyses | Conversely, no causal association between α-klotho and overall OA or knee OA was observed. |
|  | e) | Consider additional plots to visualize results (e.g., leave-one-out analyses) | The results of all sensitivity analyses are presented in the Supplementary Figure S1. |
|  | **DISCUSSION** |  |  |
| 14 | **Key results** | Summarize key results with reference to study objectives | MR studies have further highlighted a significant protective effect of increased serum α-klotho concentration specifically in individuals with the hip OA subtype. |
| 15 | **Limitations** | Discuss limitations of the study, taking into account the validity of the IV assumptions, other sources of potential bias, and imprecision. Discuss both direction and magnitude of any potential bias and any efforts to address them | However, our study also exhibits several limitations |
| 16 | **Interpretation** |  |  |
|  | a) | Meaning: Give a cautious overall interpretation of results in the context of their limitations and in comparison with other studies | However, our study also exhibits several limitations |
|  | b) | Mechanism: Discuss underlying biological mechanisms that could drive a potential causal relationship between the investigated exposure and the outcome, and whether the gene-environment equivalence assumption is reasonable. Use causal language carefully, clarifying that IV estimates may provide causal effects only under certain assumptions | our MR analysis only considered a linear relationship between exposure and outcome, thereby failing to account for potential nonlinear relationships |
|  | c) | Clinical relevance: Discuss whether the results have clinical or public policy relevance, and to what extent they inform effect sizes of possible interventions | Finally, our study focused solely on investigating the association between serum α-klotho and OA susceptibility, without delving into the specific underlying mechanisms. Further basic research and animal experiments are warranted to elucidate these mechanisms in greater detail. |
| 17 | **Generalizability** | Discuss the generalizability of the study results (a) to other populations, (b) across other exposure periods/timings, and (c) across other levels of exposure | our MR analyses exclusively utilized data from individuals of European ancestry, limiting the generalizability of our conclusions solely to this demographic group. Future MR analyses should incorporate GWAS data from diverse racial backgrounds to enhance the applicability of findings across populations |
|  | **OTHER INFORMATION** |  |  |
| 18 | **Funding** | Describe sources of funding and the role of funders in the present study and, if applicable, sources of funding for the databases and original study or studies on which the present study is based | This work was not funded by the Funding. |
| 19 | **Data and data sharing** | Provide the data used to perform all analyses or report where and how the data can be accessed, and reference these sources in the article. Provide the statistical code needed to reproduce the results in the article, or report whether the code is publicly accessible and if so, where | All data are publicly available. |
| 20 | **Conflicts of Interest** | All authors should declare all potential conflicts of interest | The authors declare no competing interests. |

This checklist is copyrighted by the Equator Network under the Creative Commons Attribution 3.0 Unported (CC BY 3.0) license.

1. Skrivankova VW, Richmond RC, Woolf BAR, Yarmolinsky J, Davies NM, Swanson SA, et al. Strengthening the Reporting of Observational Studies in Epidemiology using Mendelian Randomization (STROBE-MR) Statement. JAMA. 2021;under review.

2. Skrivankova VW, Richmond RC, Woolf BAR, Davies NM, Swanson SA, VanderWeele TJ, et al. Strengthening the Reporting of Observational Studies in Epidemiology using Mendelian Randomisation (STROBE-MR): Explanation and Elaboration. BMJ. 2021;375:n2233.
