# Supplementary material for: α-klotho reduces susceptibility to osteoarthritis: evidence from cross-sectional studies and Mendelian randomization
Source: Front Endocrinol (Lausanne). 2024 Nov 19;15:1450472. doi: 10.3389/fendo.2024.1450472 (PMC11611571; doi:10.3389/fendo.2024.1450472)

Supplementary Figure

**α-klotho reduces susceptibility to osteoarthritis: evidence from cross-sectional studies and Mendelian randomization**

**Zhao li^1^, Zhong Li^1^, Qisheng Cheng^2^, Xinlin Nie^2^, Yu Cui^1^, Bing Du^1^, Yibo Xu^1*^, Zhong Li^1*^,Teng Ma^1^**

**Figure S1.** IVW estimates of significant results from klotho on OA. (a) Scatter plots from genetically predicted klotho on OA; (b) Leave-one-out plot from genetically predicted klotho on OA; (c) Funnel plots from genetically predicted klotho on OA.


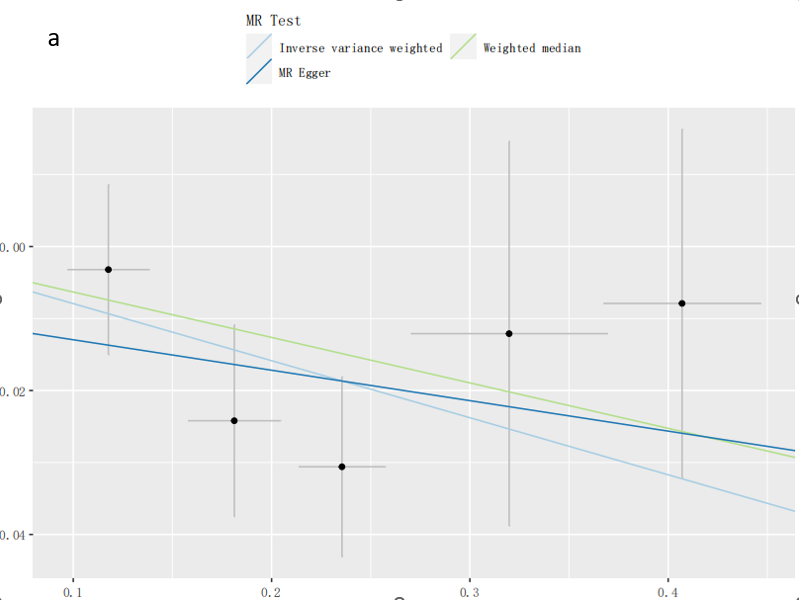


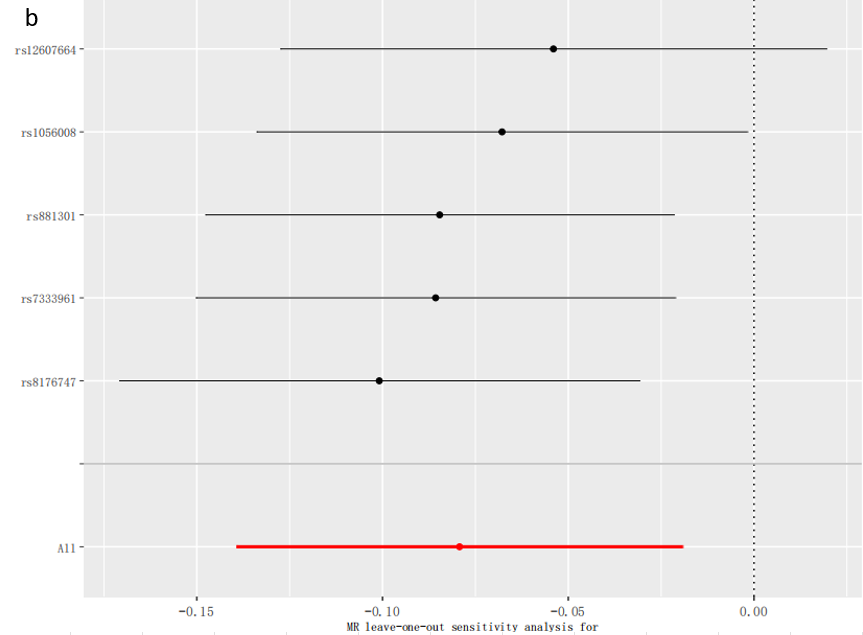

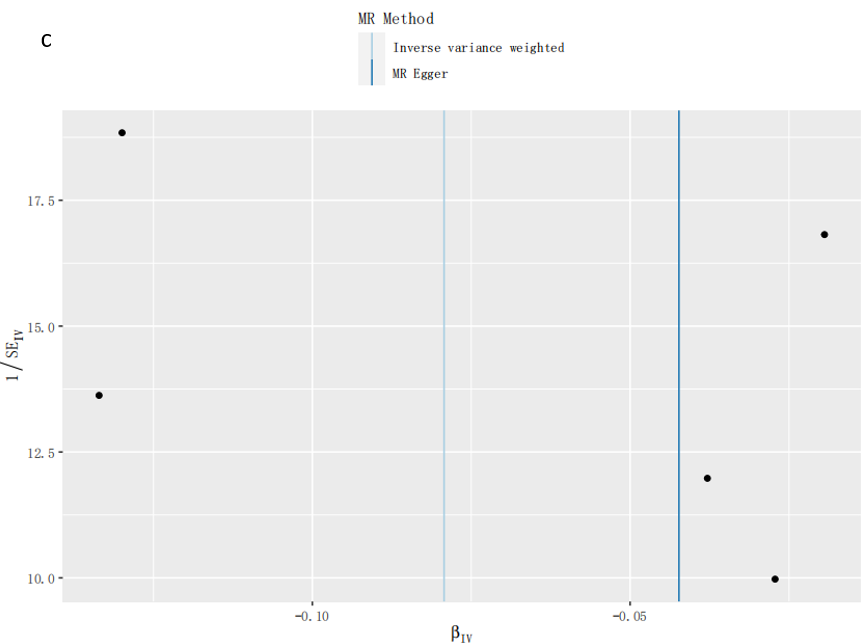

Supplement: Supplementary file 2 [file DataSheet2.docx]
